# Effect of acupuncture for patients with knee osteoarthritis: study protocol for a double-dummy randomized controlled trial

**DOI:** 10.1186/s13018-023-04198-2

**Published:** 2023-10-17

**Authors:** Shuai Yin, Yiniu Chang, Xiuli Yan, Xiaodong Feng, Nan Wu

**Affiliations:** 1 Rehabilitation Center, The First Affiliated Hospital of Henan University of Chinese Medicine, Zhengzhou, Henan China; 2 School of Medical Technology and Engineering, Zhengzhou Railway Vocational and Technical College, Zhengzhou, Henan China; 3 School of Rehabilitation Medicine, Henan University of Chinese Medicine, Zhengzhou, Henan China

**Keywords:** Knee osteoarthritis, Acupuncture, Pain, Randomized controlled trial

## Abstract

**Background:**

Acupuncture has been used to relieve chronic pain in patients with knee osteoarthritis (KOA), but the evidence is contradictory. Therefore, we carefully designed a double-dummy randomized controlled trial (RCT) to explore the therapeutic effect of acupuncture for KOA.

**Methods/design:**

A total of 138 eligible participants with KOA who consent to participate will be randomly divided into Groups A, B, and C in a ratio of 1:1:1. Participants in Group A will receive verum acupuncture and placebo gel, while those in Groups B and C will be treated with diclofenac diethylammon gel and sham acupuncture, sham acupuncture and placebo gel, respectively. The patients will receive 4 weeks of treatment, five times a week, including acupuncture treatment once a day for 30 min and gel treatment three times a day. The primary outcome will be the change of Western Ontario and McMaster Universities Osteoarthritis Index (WOMAC) at week 4. The secondary outcomes will include visual analog scale (VAS), Arthritis Quality of Life Measurement Scale Simplified Scale (AIMS2-SF), Beck Anxiety Inventory (BAI), Beck Depression Inventory (BDI) and Credibility/Expectancy Questionnaire. The evaluation will be performed at baseline, week 4, 8, and 12 after randomization.

**Discussion:**

This double-dummy RCT used diclofenac diethylammon gel as a positive control, and the completion of this trial will provide detailed and accurate evidence of the efficacy and safety of acupuncture for KOA.

*Trial registration*: China Clinical Trials Registry No.ChiCTR2100043947. Registered on September 24, 2020. https://www.chictr.org.cn/showproj.html?proj=122536.

## Introduction

Knee osteoarthritis (KOA) is a common degenerative disease of the knee joint, which is characterized by subchondral bone sclerosis, articular cartilage degeneration, periarticular bone hyperplasia, synovial lesions, and joint capsule contracture [1]. However, the precise etiology of KOA remains unclear, which may be related to obesity, gut microbiome [2], local musculoskeletal injury of knee joint [3], and so on. An epidemiological survey shows that in the USA, about 240 people per 100,000 people are diagnosed with KOA each year [4]. The prevalence of KOA in Italy, Greece, and Spain was 5.4%, 6.0%, and 12.2%, respectively [5]. The KOA is not only detrimental to the physical function of the patients and their quality of life, but also creates a huge economic burden for society as well [6, 7]. This degenerative disease will gradually worsen with age, and now, KOA has become one of the major health risks for middle-aged and elderly individuals [8].

Patients suffering from KOA are usually limited in their daily activities due to intermittent pain, weight-bearing pain, joint stiffness, and limited mobility. Patients with KOA suffer from knee pain that negatively affects their quality of life, which is one of the main reasons for them to seek medical treatment [9]. Currently, the treatment of KOA aims to relieve pain and improve knee function, so as to improve the patient's quality of life [10]. At present, there are many clinical treatments for KOA, including drug, physiotherapy, lifestyle modifications and other conservative treatments, as well as surgical treatment. However, drug treatment usually has some side effects and gastrointestinal adverse reactions [11], and the surgical treatment is expensive and frequently associated with contraindications and complications, which limit its wide application [12]. Therefore, more and more KOA patients tend to choose complementary and alternative treatment, such as manual therapy [13], acupuncture, and physical exercise [14].

Since ancient Chinese times, acupuncture has been used as a complementary and alternative treatment for KOA [15, 16]. Systematic reviews of randomized controlled trials (RCTs) have shown that acupuncture is indeed beneficial for KOA [15, 17]. In addition, studies have shown that acupuncture is more effective in treating KOA than other physical therapies such as pulsed electrical stimulation, aerobic exercise, and muscle strengthening exercise [18]. However, some guidelines stated that there was insufficient evidence to recommend acupuncture for KOA patients [19, 20]. Additionally, some research reports show that although patients report less pain, the improvement in verum acupuncture over sham acupuncture is not clinically significant [21, 22]. So, we decided to conduct an RCT to explore whether acupuncture is beneficial to KOA patients.

Topical non-steroidal anti-inflammatory drugs (NSAIDs) are another method for clinical treatment of KOA. Current management guidelines recommend topical NSAIDs as first-line therapy for KOA, especially in elderly patients [19, 23]. Topical NSAIDs are nearly as effective as oral NSAIDs in analgesia, improving physical function, and reducing stiffness, with fewer systemic adverse reactions [24, 25]. Moreover, clinical RCTs have also demonstrated the efficacy of diclofenac diethylammon gel in the treatment of KOA [26–28]. Therefore, we carefully designed this double-dummy RCT and used diclofenac diethylammon gel as a positive control, to explore the therapeutic effect of acupuncture for KOA patients.

## Methods and analysis

### Study design

**Fig. 1:**
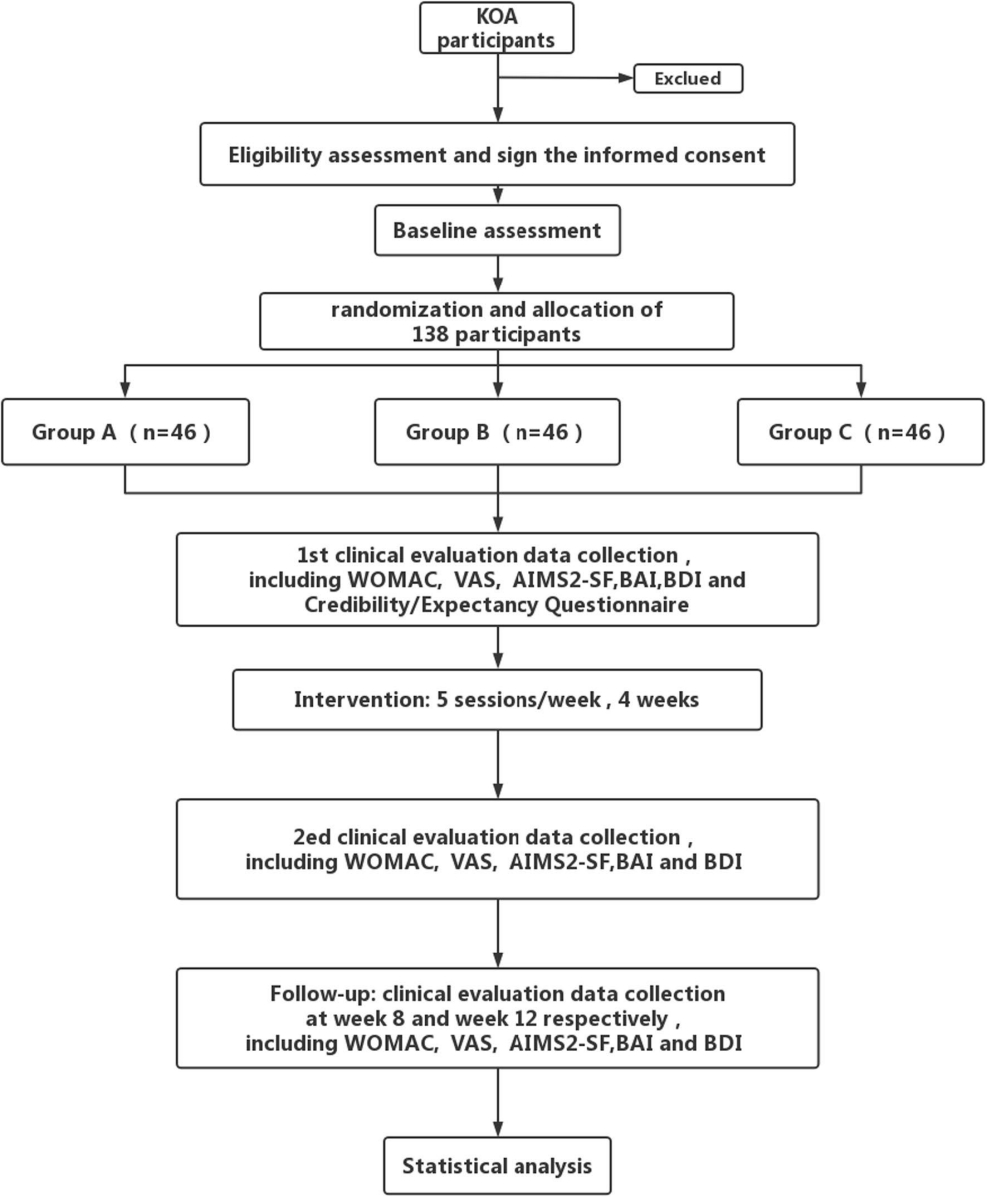
Flowchart of the trial

This is a double-dummy RCT that aims to compare the analgesic efficacy of acupuncture, diclofenac diethylammon, and placebo in the treatment of KOA, which will conduct research and write research reports in accordance with the CONSORT recommendations for reporting randomized trials. Based on clinical criteria developed by the American College of Rheumatology (ACR) [29], 138 patients with KOA will be recruited. The ratio of 1:1:1 will be used to randomize all patients after informed consent is obtained, which are Group A: verum acupuncture group (verum acupuncture and placebo gel; verum acupuncture is traditional acupuncture, a technique for treating diseases by stimulating acupoints with acupuncture); Group B: diclofenac diethylammon group (diclofenac diethylammon gel and sham acupuncture; sham acupuncture is placebo acupuncture); and Group C: placebo group (sham acupuncture and placebo gel) to receive treatments. There will be 20 sessions of treatment in total between weeks 1 and 4, with follow-ups at weeks 8 and 12. Figure 1 shows the flowchart of the trial process, and Table 1 shows the enrollment, treatment, and assessment schedule (Fig. 1 and Table 1).

**Table 1:** Study design schedule

Time point (week)	Baseline		Treatment phase			Follow-up	
	0-week	1-week	2-week	3-week	4-week	8-week	12-week
*Enrollment*							
Eligibility screen	×						
Informed consent	×						
Allocation	×						
Randomization	×						
*Interventions*							
Group A (*n* = 46)		×	×	×	×		
Group B (*n* = 46)		×	×	×	×		
Group C (*n* = 46)		×	×	×	×		
*Assessments*							
WOMAC	×				×	×	×
VAS	×				×	×	×
AIMS2-SF	×				×	×	×
BAI	×				×	×	×
BDI	×				×	×	×
Expectancy questionnaire	×						
*Participants safety*							
Adverse events	×	×	×	×	×	×	×

Qualification screening and informed consent will be completed before the assignment. After allocation, each patient will be treated within 4 weeks. The evaluation will be performed at baseline and weeks 4, 8, and 12, respectively, after randomization. Adverse events will be recorded on the case report form at any time during treatment

### Study setting and recruitment

The trial will be conducted at the First Affiliated Hospital of Henan University of Chinese Medicine. The outpatient department of the hospital will recruit 138 KOA participants. The clinical research coordinator will perform a preliminary screening of participants and provide a detailed explanation to eligible participants in detail of the purpose of the study, and written informed consent will be obtained [30]. Afterward, the clinical assessor will conduct baseline assessments of participants who have obtained written informed consent. A randomization number will be assigned to each patient at enrollment, which is a unique direct identifier included on the case report forms (CRF).

### Participants

It is required that all participants sign the informed consent document before inclusion in this trial, which outlines the trial's benefits and risks. Moreover, patients can withdraw from the study at any time without penalty or loss of benefits and without any reasons. This trial is a voluntary one, it is the patient's right to decide whether to participate in this trial, and all information about the participants will be kept confidential.

### Inclusion criteria

The inclusion criteria are as follows: (1) adults aged 45–75 years, including the age of 45 and 75, male or female; (2)patients must fulfill the standard diagnosis of KOA established by the ACR; (3) radiologic confirmation of KOA [31] (Kellgren–Lawrence grades I to III); (4) VAS score ≥ 40 mm; (5) symptoms of knee bone friction sound during activity, pain, and morning stiffness ≤ 30 min have been present for over 6 months; and (6) participants fully understand this trial and written informed consent is signed.

### Exclusion criteria

The exclusion criteria are as follows: (1) patients with knee infection; (2) patients with any ligament or meniscus tear or acute inflammation of the synovial sac; (3) patients with a history of local tumor or malignancy of the knee joint; (4) patients with physical or laboratory findings of the presence of infection, autoimmune diseases, or inflammatory arthritis; (5) patients who have received hyaluronic acid injections or corticosteroid injections within the last 3 months; (6) patients who received acupuncture or electro-acupuncture within 8 weeks prior to participating in this trial; and (7) patients suffering from pain in other body parts.

### Discontinuation criteria and modification

Participants who meet the following criteria will be excluded from the trial during the trial period: (1) receiving additional treatments that may interfere with acupuncture's efficacy, violating the protocol, such as taking analgesics without permission or receiving other treatments; (2) participants were reluctant to continue treatments; (3) missing more than 4 of 20 acupuncture treatment sessions; and (4) serious adverse events occurred and doctors consider the trial should be terminated.

### Sample size

Power Analysis and Sample Size (PASS, provided by Shanghai Datanine Software Co., Ltd., China) was used for sample size calculation. Based on prior studies and clinical experience, we expected the WOMAC total score to decrease by 17 points after acupuncture, a reduction by 13 points after diclofenac diethylammon gel, and a reduction by 10 points in the placebo group [32, 33]; the standard deviation is 10.24. The type I error inflation can be avoided by setting the significance level at 0.05 on a two-sided basis. Forty-two participants in each group will have 80% power to detect significant differences between the groups. The sample size was increased to 46 participants in each group to supplement for a possible 10% loss at follow-up. So, this trial will include 138 participants in total.

### Randomization

In this trial, an independent researcher, who will not involve in any other trial procedures, will use a computer to create a random number list and then randomly assign eligible patients in a 1:1:1 ratio to one of the three groups. Sealable envelopes will be used for randomization allocation. The evaluator will inform the acupuncturists after the participant signs the informed consent form and completes the baseline assessments. The acupuncturists will open the envelope according to the participant’s screening sequence number and assign them to different groups to receive the intervention.

### Blinding

Acupuncturists cannot be blinded as they will be required to apply the various stimulation methods to intervene. However, there will be blinded of group assignments among the participants, because the acupuncture needles are the same, and the diclofenac diethylammon gel and placebo gel are identical in appearance. In addition, participants will be arranged in different rooms for treatment, in order to prevent them from communicating with each other to understand their treatment programs, and the acupuncturists will not communicate any information to participants about their treatment programs. Group assignments will be concealed from outcome assessor and data analysts. Only after data analysis, we will perform unblinding analysis. In the data summary stage, the three separation principles of researcher, operator, and statisticians will be implemented.

### Researchers

These three groups of participants will be treated by acupuncturists registered with China’s Ministry of Health which have over 6 years of clinical experience. Moreover, the data collectors and analysts who are not aware of the random assignment protocol will monitor all phases of the trial. All researchers will receive rigorous training about project objectives and standard procedures before the start of the trial, including those responsible for recruiting test participants, outcome assessor, acupuncturists, and statisticians.

### Safety assessment

During the trial, adverse events (AEs) will be documented by using safety assessments, which include date, extent, frequency, and duration of AEs. The most common AEs related to acupuncture in this trial were pain, bleeding, hematomas, fainting, or other severe events. Moreover, redness, swelling, and itching of the skin are the AEs associated with diclofenac diethylammon gel. In the event of adverse events, emergency measures will be taken and documented in detail in the CRF. In the event of severe adverse events, the research ethics committee will determine whether the participant should be withdrawn from the study. Patients may withdraw from this trial if any AEs occur.

### Interventions

This trial is divided into three groups: Group A: verum acupuncture group (verum acupuncture and placebo gel), Group B: diclofenac diethylammon group (diclofenac diethylammon and sham acupuncture), and Group C: placebo group (sham acupuncture and placebo gel). Aside from the experimental scheme, patients will be advised not to use other any methods for the treatment of KOA. At the beginning of the trial, all licensed acupuncturists, who have at least 6 years of experience in acupuncture, will be trained in the clinical operation organized by the research group.

### Group A

**Fig. 2:**
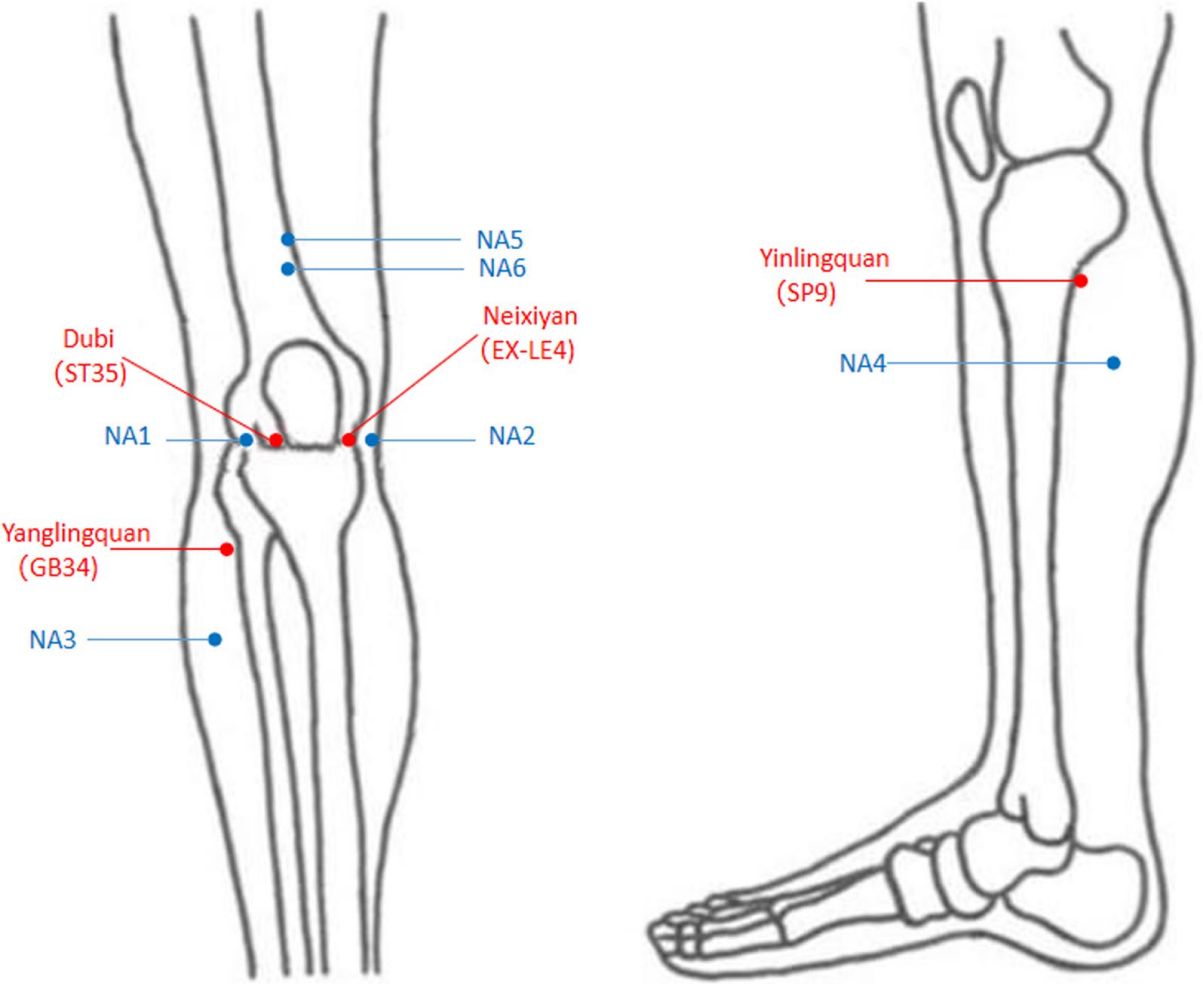
The picture of acupuncture points

Subjects in verum group will be treated with verum acupuncture and placebo gel for 4 weeks. We will determine the locations of the acupoints using the nomenclature and locations of acupuncture points specified in the National Standard of the People's Republic of China (GB/T 12346-2006) including SP9 (Yinlingquan), GB34 (Yanglingquan), EX-LE4 (Neixiyan), and ST35 (Dubi) (Table 2 and Fig. 2). Furthermore, two Ashi acupoints (where the patient feels the most pain) will be added based on patients condition.

**Table 2:** Locations of acupoints for Group A

Acupoint	Location
SP9	On the tibial aspect of the leg, in the depression between the inferior border of the medial condyle of the tibia and the medial border of the tibia
GB34	On the fibular aspect of the leg, in the depression anterior and distal to the head of the fibula
EX-LE4	On the anterior aspect of the knee, in the depression medial to the patellar ligament
ST35	On the anterior aspect of the knee, in the depression lateral to the patellar ligament

During acupuncture, the patient lies supine in a comfortable position, while the acupuncturist will sterilize the patient's skin with 75% alcohol and then use sterile needles (0.25 mm in diameter, 40 mm in length, Huatuo, Suzhou, China) to the acupoints, a depth of which is 21–26 mm. In order to generate "deqi," all needles will undergo manipulations of twirling and lifting, using a reinforcing-reducing method. The "*deqi*" refers to after the acupuncture is inserted into the acupoints, the patients may feel numbness, soreness, and heaviness, after manual manipulation or needle retention for a long time which are believed to be one of the key factors in the acupuncture efficacy. After that, the stimulation operation is performed every 10 min to maintain the *deqi* sensation, and each treatment lasts for 30 min. The verum acupuncture treatment will be continued for 4 weeks, 5 times a week, with 2 days of rest in between each week.

Placebo gel is manufactured by the Pharmacy Department of the First Affiliated Hospital of Henan University of Chinese Medicine. Its ingredients are white gel matrix, without any therapeutic effect, but it is the same as diclofenac diethylammon gel in appearance and texture. It will be applied to the surface of each affected knee three times a day for 5 consecutive days and for 4 consecutive weeks.

### Group B

In the diclofenac diethylammon group, the subjects will be treated with diclofenac diethylammon gel and sham acupuncture for 4 weeks. Diclofenac diethylammon gel (approval number H19990291, manufactured by Novartis Pharmaceutical Co., Ltd., Beijing, China, specification: 20 g. Each gram of gel contains 10 mg of diclofenac diethylammon) will be applied to the surface of each affected knee three times a day for 5 consecutive days for 4 consecutive weeks.

The acupoints for sham acupuncture will select six non-acupuncture points that are separate from traditional acupuncture or meridians. The non-acupoints are shown in Table 3 and Fig. 2. The acupuncture procedure will be the same as Group A, but in order to avoid deqi sensations as much as possible, the needle depth is 1–2 mm and do not have any manipulation, directly retained for 30 min. The sham acupuncture treatment will also last 4 weeks, 5 times a week, with 2 days of rest in between each week.

**Table 3:** Locations of non-acupoints for Groups B and C

Non-acupoint	Location
NA1	Located between ST35 and gallbladder meridian, on the same level as ST35
NA2	Located between EX-LE4 and spleen meridian, on the same level as EX-LE4
NA3	3 cun* below GB34 (between the gallbladder and bladder meridian)
NA4	3 cun* below SP9 (between the spleen and kidney meridian)
NA5	On the anterior aspect of the thigh, 5 cun above the upper edge of the patella (between the spleen and stomach meridian)
NA6	On the anterior aspect of the thigh, 4 cun above the upper edge of the patella (between the spleen and stomach meridian)

*1 cun (≈ 20 mm) is defined as the width of the interphalangeal joint of the patient’s thumb

### Group C

In the placebo group, subjects will be treated with sham acupuncture and placebo gel for 4 weeks. Sham acupuncture treatment procedure is the same as in Group B, and placebo gel will apply the same gel as in Group A.

### Outcome assessments

Outcome assessments were performed only on a knee that meets the inclusion criteria; when both knees meet the inclusion criteria, the more painful knee will be assessed.

### Primary outcome

The Western Ontario and McMaster Universities Osteoarthritis Index (WOMAC) [34] will be the primary outcome measure for this trial. The WOMAC is an osteoarthritis questionnaire with high reliability and validity that has been widely used in various clinical trials involving KOA patients [35]. WOMAC includes 5 pain-related items, 2 stiffness-related items, and 17 physical function-related items, each item is scored on a scale of 0–10, and the total score is on a scale of 0–240, with higher scores indicating more severe KOA symptoms in patients [36]. Outcome assessor will assess participants at baseline, week 4, week 8 and week 12, but the primary outcome will be the assessment at week 4.

### Secondary outcomes

1. The WOMAC subscale (pain, stiffness, and physical function) at baseline, week 4, week 8, and week 12 and the total WOMAC scores at week 4 and week 8 will be used as secondary outcome indicators.
2. Pain visual analog scale (VAS) [37]: Using a 100-mm horizontal line anchored by two descriptors, patients will determine the most relevant point for quantifying their pain intensity: a score of 0 represents “no pain” and a score of 100 represents “severe pain.” After randomization, VAS will be collected at baseline, week 4, week 8, and week 12.
3. Arthritis Quality of Life Measurement Scale Simplified Scale (AIMS2-SF) [38] includes five dimensions of physical function, pain symptoms, influence, social interaction, and role components, with a total of 26 items, a scale of 1–5 is applied to each item, and the sum of all five dimensions represents the total score. After randomization, the AIMS2-SF will be collected at baseline, week 4, week 8, and week 12.
4. Credibility/Expectancy Questionnaire [39] is a measure of treatment expectations and reasonable confidence for use in clinical outcome studies and it is only assessed once at the baseline.
5. Emotional Monitoring: Patients with KOA are often accompanied by symptoms of anxiety and depression. In this trial, patients' emotional states will be assessed using the Beck Anxiety Inventory (BAI) [40] and Beck Depression Inventory (BDI) [41]. After randomization, BAI and BDI will be collected at baseline, week 4, week 8, and week 12.

### Data collecting and monitoring

In this trial, all participants' data will be managed in the CRF both on paper and electronically. In order to ensure consistency of source data, all scales will be assessed by the same outcome assessor. Two researchers will independently enter the data into the EPIDATA electronic database to ensure accuracy. The Center for Evidence-Based Medicine will monitor the study and data every 3 months [42].

### Statistical analysis

The statistical analysis procedure of this trial will be performed using SPSS 22.0 software (IBM Corporation, Armonk, NY, USA). Statisticians responsible for data analysis will be independent of the research team and blind to the test settings. The data will be tested for normality using the quantile–quantile plots. We will analyze the data from WOMAC, VAS, AIMS2-SF, BAI, and BDI within the group and use paired-sample t-tests for those data that follow normal distributions, and in the case of data that did not conform to the normal distribution, Wilcoxon test will be used. Age, height, weight, and outcome assessments will be compared among the three groups, the one-way analysis of variance (ANOVA) will be applied to data conforming to a normal distribution, and the Kruskal–Wallis one-way ANOVA will be used to analyze data that do not follow a normal distribution. Intention-to-treat analysis will be used for outcome measurements to analyze missing values, based on the last observation carried forward method. In the analysis of missing values, only patients who attended at least 80% of the sessions and completed the follow-up in the allocated intervention group will be included. In addition, data will be tested by bilateral tests, and the confidence intervals are all 95% bilateral; *p* < 0.05 (*α* = 0.05) is considered to indicate statistical significance.

### Patient and public involvement

In the development of this clinical trial protocol, patients and the general public will not have any involvement.

## Discussion

Based on published systematic reviews and meta-analyses of the RCTs, acupuncture seems to be a promising for the treatment of KOA [12]. However, due to the lack of appropriate blind methods and control group settings, the existing experimental studies are of low methodological quality and therefore cannot be used as clinical evidence to verify the efficacy of acupuncture. So, this trial was designed as a double-dummy RCT; as a result of the completion of this trial, detailed and accurate evidence will be available regarding acupuncture's efficacy and safety in treating KOA. The following are some of the advantages of the trial design.

### Double-dummy design can effectively improve the experimental design quality

Double dummy is a common technique in clinical trials. In clinical trials, when the two treatments are inconsistent in appearance or pathway, a placebo is prepared for each treatment group and control group in order to achieve blinding and thus the treatment group and the control group in the appearance or approach to maintain the same treatment. Double-dummy technology is used to realize the blind method, and the acupuncture clinical research model with acupuncture therapy and medicine as control can be formed, which can improve the quality of acupuncture clinical trials. At present, some studies also have adopted the double-dummy design in the treatment of acupuncture for migraine and insomnia, which achieved the purpose of double-dummy patients by designing verum acupuncture and placebo versus sham acupuncture and true medicine [43, 44]. In this study, according to the double-dummy design, all three groups will receive verum/sham acupuncture combined with diclofenac diethylammon/placebo gel treatment. In order to minimize the potential therapeutic effect and gain high patient trust from patients, we will carefully take into consideration the choice of sham acupuncture and placebo.

However, it is difficult to design an inert placebo control for acupuncture since it is a physical intervention. Currently, acupuncture at ineffective acupoints or non-effective acupuncture at effective acupoints is used in KOA clinical trials as a placebo-controlled approach. It is possible, however, that these approaches may result in a wide range of physiological responses [45, 46]. Therefore, our placebo acupuncture method will consist of non-effective acupoints with the non-effective puncture at the local of the knee as the placebo acupuncture. Moreover, after needle insertion, acupuncturists will be requested not to perform any further manipulation to avoid deqi sensation and minimize the possible effects of sham acupuncture. In addition, the placebo gel is identical in appearance, odor, dosage, color, and texture to the diclofenac diethylammon gel, but has no other substance that might affect efficacy assessments.

### The selection of acupoint and non-acupoint is reasonable

As we know, acupoints can treat diseases in the local and surrounding areas where they are located [47]. In this trial, four acupoints around the knee joint will be used for verum acupuncture, including SP9, GB34, EX-LE5, and ST35, which are mostly located around knee joint on ligaments and tendons. At the same time, two *Ashi* acupoints will be selected according to the syndrome type to fulfill the needs for individualized treatment, which are effective in relieving local pain.

In sham acupuncture, six non-acupoints are also selected around the knee joint. These non-acupoints are separate from conventional acupoints or meridians, which may allow patients receiving sham acupuncture to overcome psychological bias.

In this trial, we will set a standardized acupuncture treatment program and manipulation, which purpose is to evaluate the therapeutic effects of acupuncture, a drug, and a placebo on KOA. As a result of the completion of this trial, evidence for acupuncture to relieve pain and improve physical function in KOA patients can be provided, as well as a reference for the clinical application of acupuncture treatment of KOA.

### Trial status

The recruitment of patients for this study has not yet begun. The recruitment procedure will begin on October 1, 2023. The authors expect to complete this procedure on December 31, 2024. The protocol (version 2) has been registered at China Clinical Trials Registry on September 24, 2020 (No. ChiCTR2000038554).

## Data Availability

Not applicable. The manuscript does not contain any data.

## Abbreviations

KOA: Knee osteoarthritis
RCT: Randomized controlled trial
ACR: American College of Rheumatology
WOMAC: Western Ontario and McMaster Universities Osteoarthritis index
AIMS2-SF: Arthritis Quality of Life Measurement Scale Simplified Scale
VAS: Visual analog scale
CRF: Case report forms
